# Lee mortality index as comorbidity measure in patients undergoing radical cystectomy

**DOI:** 10.1186/s40064-015-0834-9

**Published:** 2015-02-03

**Authors:** Michael Froehner, Rainer Koch, Vladimir Novotny, Ulrike Heberling, Stefan Propping, Rainer J Litz, Matthias Hübler, Gustavo B Baretton, Oliver W Hakenberg, Manfred P Wirth

**Affiliations:** Department of Urology, University Hospital “Carl Gustav Carus” Technische Universität Dresden, Fetscherstrasse 74, Dresden, D-01307 Germany; Department of Medical Statistics and Biometry, University Hospital “Carl Gustav Carus” Technische Universität Dresden, Fetscherstrasse 74, Dresden, D-01307 Germany; Department of Anesthesiology, University Hospital “Carl Gustav Carus” Technische Universität Dresden, Fetscherstrasse 74, Dresden, D-01307 Germany; Department of Pathology, University Hospital “Carl Gustav Carus” Technische Universität Dresden, Fetscherstrasse 74, Dresden, D-01307 Germany; Department of Anesthesiology, University Hospital Bergmannsheil, Bürkle-de-la-Camp-Platz 1, Bochum, 44789 Germany; Department of Urology, University of Rostock, Ernst-Heydemann-Strasse 6, Rostock, D-18055 Germany

**Keywords:** Urologic neoplasms, Comorbidity, Mortality, Bladder, Cystectomy, Proportional hazards model

## Abstract

To investigate the recently described Lee mortality index as predictor of mortality after radical cystectomy.

A total of 735 patients who underwent radical cystectomy for bladder cancer between 1993 and 2010 were studied. Median patient age was 67 years and the median follow-up was 7.8 years (censored patients). The Lee mortality index was assigned based on data derived from patient history, preoperative cardiopulmonary risk assessment and discharge records. The age-adjusted Charlson score and preoperative cardiopulmonary risk assessment classifications were used for comparison. Competing risk analysis and Cox proportional hazard models for competing risks were used for the statistical analysis.

The Lee mortality index predicted competing mortality in a dose–response relationship with somewhat lower 10-year mortality rates than predicted (p = 0.0120). Beside the age-adjusted Charlson score, the Lee mortality index was an independent predictor of overall mortality (hazard ratio per unit increase 1.06, p = 0.0415) and replaced the age-adjusted Charlson score as predictor of competing mortality (hazard ratio (HR) per unit increase 1.27, p < 0.0001). The American Society of Anesthesiologists (ASA) physical status classification was also an independent predictor of overall (HR for ASA 3–4 versus 1–2: 1.53, p = 0.0002) and competing mortality (HR for ASA 3–4 versus 1–2: 1.62, p = 0.0044).

The Lee mortality index is a promising and easily applicable tool to predict competing mortality after radical cystectomy. It is at least equal to the age-adjusted Charlson score and may be supplemented by information provided by the ASA classification.

**Electronic supplementary material:**

The online version of this article (doi:10.1186/s40064-015-0834-9) contains supplementary material, which is available to authorized users.

## Introduction

Age, performance status and comorbidities are important factors influencing treatment choice and outcome in patients with muscle-invasive bladder cancer. Radical cystectomy usually is the treatment of choice, although several approaches with different aggressiveness and side effect profiles are in use. However, randomized trials favoring one approach other the others are largely lacking. Predicting competing mortality more precisely might help to decide when to opt for bladder-preserving treatment alternatives in this patient population (Koppie et al. [2008]; Witjes et al. [2014]). Currently, the guidelines on muscle-invasive and metastatic bladder cancer of the European Association of Urology (EAU) (Witjes et al. [2014]) recommend using the age-adjusted Charlson score (Charlson et al. [1994]) for this purpose. However, there is no gold standard for comorbidity assessment in cancer patients (Sarfati [2012]). Recently, the easily applicable Lee mortality index predicting 10-year mortality rates was developed and validated in a nationally representative sample of community-dwelling US citizens older than 50 years (Lee et al. [2006]; Cruz et al. [2013]). In this study, we tested the ability of this index to stratify patients undergoing radical cystectomy for bladder cancer according to their risk of competing mortality.

## Patients and methods

### Patient sample

The study sample consisted of 735 patients with complete histopathological and comorbidity data (out of 796 consecutive patients) who had undergone radical cystectomy for muscle-invasive or high risk non-muscle-invasive urothelial or undifferentiated carcinoma of bladder at our institution between 1993 and 2010. Institutional review board approval was obtained. 53% of patients had organ-confined, node-negative disease and 26% had lymph node metastases. Continent urinary diversion was performed in 38% of cases (Froehner et al. [2014a]).

### Data collection

The Lee mortality index was developed and validated in the Health and Retirement Study population, a nationally representative sample of community-dwelling US citizens older than 50 years (Lee et al. [2006]; Cruz et al. [2013]). In this mortality index, risk points are given for age, male sex, current tobacco use, body mass index <25 kg/m^2^, diabetes mellitus, non-skin cancers, chronic lung disease, congestive heart failure and four functional categories (Table 1). We made minor modifications in order to adapt it to the available data without stratifying between skin and non-skin cancer, disregarding the functional impairments not available in our database and classifying all documented cases of heart failure as congestive. Bladder cancer itself was not used for assigning the Lee mortality index risk points and competing mortality (i. e. mortality from other causes than uncontrolled bladder cancer progression) was used as end point for comparison of the observed with the predicted 10-year mortality rates (Cruz et al. [2013]). Comorbidity information was obtained from patient history, preoperative cardiopulmonary risk assessment and discharge records. The following variables were included for multivariate analysis: age (continuous variable), Lee mortality index (continuous variable), age-adjusted Charlson score (Charlson et al. [1994]) (continuous variable), American Society of Anesthesiologists (ASA) physical status classification (American Society of Anesthesiologists [2015]) (1 versus 2 versus 3–4 and 1–2 versus 3–4), New York Heart Association classification of cardiac insufficiency (The Criteria Committee of the New York Heart Association [1994]) (0 versus 1 versus 2+), (Canadian Cardiovascular Society [2015]) (0 versus 1 versus 2+), body mass index (continuous variable), tumor stage (localized, node-negative versus locally advanced node-negative versus node positive), lymph node density (continuous variable), number of removed lymph nodes (<10 versus 10–20 versus 20+), number of involved lymph nodes (0 versus 1 versus 2+), adjuvant cis-platin-based chemotherapy (no versus yes), urinary diversion (continent versus incontinent or none).

**Table 1:** **Parameters and corresponding weights constituting the Lee mortality index** (Lee et al. [2006])

Parameter	Weight (points)
Age 60–64 years	1
Age 65–69 years	2
Age 70–74 years	3
Age 75–79 years	4
Age 80–84 years	5
Age 85+ years	7
Male sex	2
Current tobacco use	2
Body mass index <25 kg/m^2^	1
Diabetes mellitus	1
Malignant tumor (excluding minor skin cancers)	2
Chronic lung disease	2
Congestive heart failure	2
Difficulties with bathing*	2
Difficulties with managing finances*	2
Difficulties with walking several blocks*	2
Difficulties with pulling or pushing larger objects*	1

The index is calculated by adding the points an individual patient gained. The parameters indicated with asterisk were not assessed in this study.

### Statistical analysis

Competing risk analysis and Cox proportional hazard models for competing risks were used for the statistical analysis which was done with the Statistical Analysis Systems V9.4 statistical package (SAS Institute, Cary, NC).

## Results

**Figure 1:**
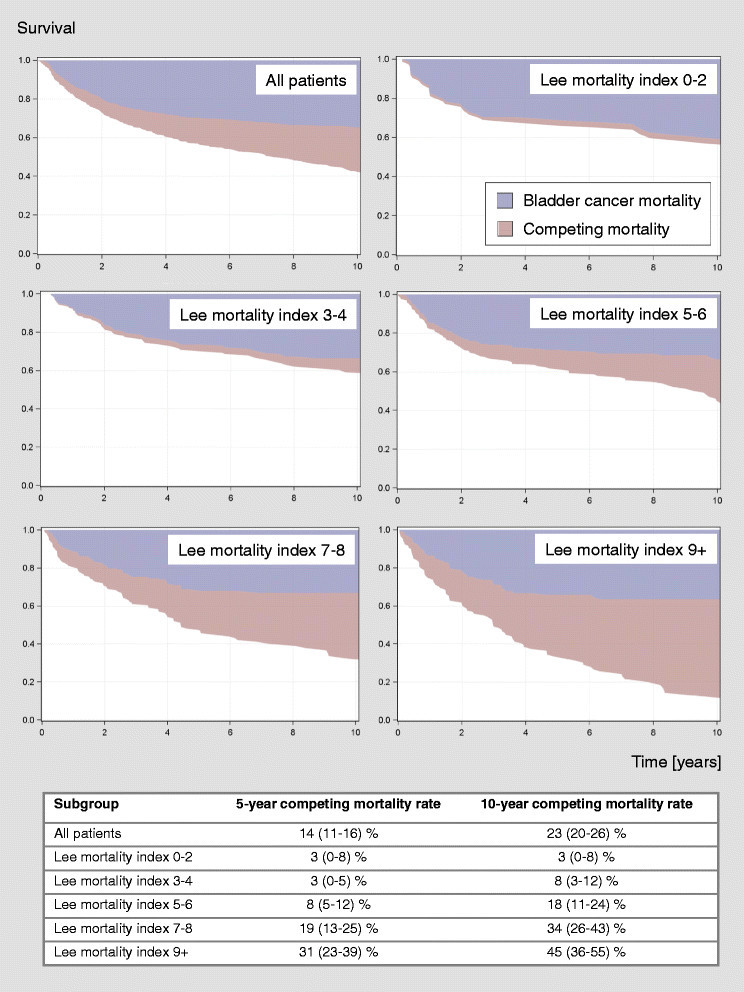
**Competing risk analysis stratified by the modified Lee mortality index illustrating the dose**–**response relationship between the Lee mortality index and competing mortality and the absence of a relationship with bladder cancer mortality after radical cystectomy.** Five- and ten-year competing mortality rates with 95% confidence intervals for the different Lee mortality index subgroups are shown in the table below.

The median age was 67 years and the median follow-up of the censored patients was 7.8 years. The patients were distributed relatively evenly over a wide range of Lee mortality index classes (0–13, Table 2). The Lee mortality index predicted competing mortality after radical cystectomy in a risk-effect relationship (Figure 1) whereby the observed 10-year mortality rates were somewhat lower than predicted by the index (Table 2). In multivariate analysis the Lee mortality index maintained independent prognostic significance beside the age-adjusted Charlson score when overall mortality was considered and completely replaced the age-adjusted Charlson score when competing mortality was considered (Table 3). When all non-significant parameters were included into the model with competing mortality as the endpoint, the p value for the age-adjusted Charlson score was 0.8841. Of all the other investigated comorbidity measures, only the ASA classification was also an independent predictor of overall and competing mortality (Table 3).

**Table 2:** **10-year competing mortality rates after radical cystectomy stratified by the Lee mortality index compared with the predicted values** (Cruz et al. [2013])

Points	Predicted 10-year overall mortality(Cruz et al.[2013])	95% CI(Cruz et al.[2013])	Proportion of events*	Observed 10-year competing mortality	95% CI	p**
**0**	2.8%	1.3-4.2%	0/8	0.0%	NA	0.0002
**1**	4.0%	2.6-5.4%	1/12	9.4%	0.0-30.7%	0.4923
**2**	6.0%	4.8-7.3%	2/54	1.9%	0.0-5.6%	0.0088
**3**	9.1%	7.6-11%	4/59	3.5%	0.0-8.3%	0.0144
**4**	14%	12-16%	14/118	9.5%	2.7-16.3%	0.2134
**5**	21%	19-23%	20/109	17.8%	9.2-26.4%	0.4775
**6**	30%	27-33%	21/103	25.0%	13.9-36.1%	0.3940
**7**	40%	36-43%	36/97	36.0%	23.9-48.0%	0.5321
**8**	52%	48-55%	22/57	32.3%	17.4-47.1%	0.0114
**9**	62%	58-66%	22/52	49.8%	24.1-75.6%	0.3588
**10**	71%	67-76%	13/35	40.8%	19.7-61.8%	0.0060
**11**	81%	76-85%	12/21	55.6%	21.8-73.5%	0.0578
**12**	85%	81-90%	2/8	25.0%	0.0-52.1%	<0.0001
**13**	89%	85-94%	2/2	100%	NA	<0.0001
**14+**	95%	93-98%	0/0	NA	NA	NA

With an overall p value of 0.0120, the observed competing mortality rates rated were somewhat lower than predicted. CI: confidence interval, NA: not available, *deaths of competing causes (other than bladder cancer) per number of patients in this risk group, **p values are raw values.

**Table 3:** **Optimal Cox proportional hazard models predicting overall (upper part) and competing mortality (lower part) after radical cystectomy**

Endpoint overall mortality			
Category	Hazard ratio	95% confidence interval	p
Lee mortality index (continuous variable, per unit increase)	1.06	1.00-1.12	0.0415
Age-adjusted Charlson score (continuous variable, per unit increase)	1.08	1.02-1.15	0.0100
Locally advanced, lymph node-negative disease	2.23	1.72-2.89	<0.0001
Lymph node-positive disease	5.41	4.09-7.15	<0.0001
10-20 lymph nodes removed	0.82	0.63-1.08	0.1614
>20 lymph nodes removed	0.72	0.52-0.98	0.0340
ASA 2	9.66	1.35-69.27	0.0274
ASA 3-4	14.35	2.00-103.76	0.0083
Adjuvant cisplatin-based chemotherapy	0.53	0.40-0.70	<0.0001
**Endpoint competing mortality**			
**Category**	**Hazard ratio**	**95% confidence interval**	**P**
Lee mortality index (continuous variable, per unit increase)	1.27	1.19-1.35	<0.0001
ASA 3-4	1.62	1.62-2.26	0.0044
Locally advanced, lymph node-negative disease	1.31	0.92-1.87	0.1313
Lymph node-positive disease	0.54	0.35-0.85	0.0071

In the analysis of competing mortality, the ASA classes 1 and 2 were combined since in the 15 patients with ASA class 1 no competing death occurred up to now prohibiting the use of this category as reference. Combining the ASA classes 1 and 2 did not change the optimal model predicting overall mortality meaningfully (hazard ratio for ASA 3–4 versus 1–2: 1.53, p = 0.0002). The inverse relationship between lymph node involvement and competing mortality may be explained by the clear overweight of bladder cancer mortality in this subgroup. Reference categories: organ confined, lymph node-negative disease, <10 lymph nodes removed, ASA 1 or ASA 1–2, respectively, no adjuvant cisplatin-based chemotherapy or unknown (n = 10).

## Discussion

In this study, the Lee mortality index was at least equal to the age-adjusted Charlson score as predictor of competing mortality after radical cystectomy. The observed mortality rates were somewhat lower than the predicted values based on a nationally representative US sample. This difference could possibly be explained by an elimination of extremely unfit patients by preoperative selection and a more critical evaluation of the health status prior to major surgery also capturing more minor conditions compared to a questionnaire-based approach (Lee et al. [2006]; Cruz et al. [2013]). Compared to more strictly selected patients undergoing radical prostatectomy (Froehner et al. [2014b]), the difference between the predicted and the actually observed mortality rates was somewhat lower in the radical cystectomy setting (accounting for approximately one Lee mortality index risk point compared to approximately two risk points in the radical prostatectomy setting (Froehner et al. [2014b])).

The current EAU guidelines on muscle-invasive and metastatic bladder cancer recommend using the age-adjusted Charlson score for estimating comorbidity and selecting patients for radical cystectomy (Witjes et al. [2014]). The Charlson score assesses a panel of conditions with different weights of severity and adds one point for each life decade of age over 40 years (Charlson et al. [1994]). Compared with the age-adjusted Charlson score (Charlson et al. [1994]), the Lee mortality index (Lee et al. [2006]) weights age in a somewhat different way, is based on less parameters (making it simpler to use), weights these conditions differently and includes several functional parameters (which were, however, not included in this study). The availability of relatively contemporary mortality figures (enrolment year 1998 (Lee et al. [2006]; Cruz et al. [2013])) in a large population-based sample is a possible advantage of the Lee mortality index. In contrast, the age-adjusted Charlson score was developed with patients recruited between 1982 and 1985 (Charlson et al. [1994]). It is conceivable that the prognostic weights of some of the contributing factors have changed in the meantime.

The supplementary prognostic impact of the ASA classification in the models predicting overall and competing mortality, respectively, suggests that further prognostic information may be derived from this classification (Table 1). It is an alternative to simply counting and weighing concomitant diseases and seems to be a reasonable approach for assessing the general health status. It has been identified as a predictor both of the short-term (Aziz et al. [2014]; Boorjian et al. [2013]) and the long-term mortality after radical cystectomy (Boorjian et al. [2013]; Mayr et al. [2012]). In view of this data, the statement of the current EAU guidelines regarding the ASA classification with respect to candidates for radical cystectomy (“…does not address comorbidities and should not be used in this setting.” (Witjes et al. [2014])) should be critically reviewed in view of this data.

Several limitations of this study have to be addressed. No data on the functional status categories assessed by the Lee mortality index was available. Although the incidence of these functional impairments may be expected to be low in this patient population, it is not entirely clear in which degree these parameters would influence the results by inflating the higher risk classes. Including this information would likely increase the difference between the predicted and observed mortality figures (Table 2). Using the preoperative cardiopulmonary risk assessment as a data source might capture minor health impairments that would be missed in a questionnaire study and might dilute the risk classes of the Lee mortality index. Because of the higher contribution of bladder cancer to overall mortality (Figure 1) the comparison of the competing mortality rates with the overall mortality rates predicted by the Lee mortality index (Table 2) should be interpreted with some caution.

## Conclusion

The Lee mortality index is a promising tool to predict competing mortality after radical cystectomy. It is at least equal to the age-adjusted Charlson score and may be supplemented by information gained from the ASA classification.

### Ethical standards

Institutional review board approval was obtained (reference number: EK84032009).

## Authors’ original submitted files for images

Below are the links to the authors’ original submitted files for images. Authors’ original file for figure 1
