# Supplementary figures and images for: Lee mortality index as comorbidity measure in patients undergoing radical cystectomy
Source: Springerplus. 2015 Feb 3;4(1):55. doi: 10.1186/s40064-015-0834-9 (PMC4320229; doi:10.1186/s40064-015-0834-9)

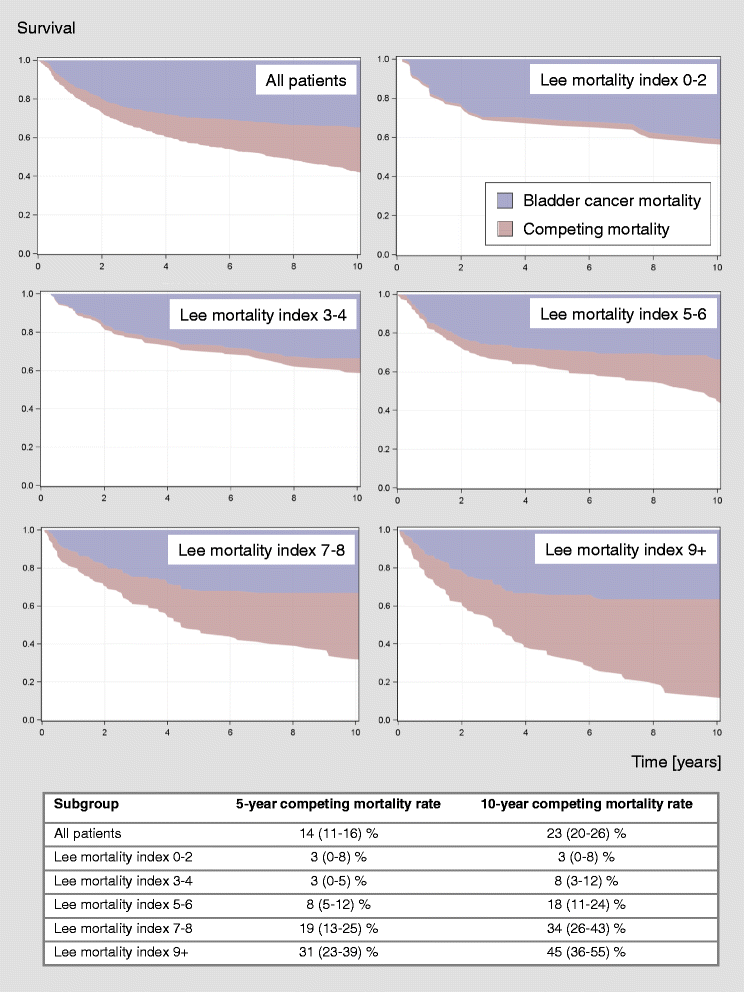

Supplement: Supplementary file 1 — Authors’ original file for figure 1 [file 40064_2015_834_MOESM1_ESM.gif]
